# The Decline of Freedom of Expression and Social Vulnerability in Western democracy

**DOI:** 10.1007/s11196-023-09990-1

**Published:** 2023-03-12

**Authors:** Aniceto Masferrer

**Affiliations:** University of Valencia, Valencia, Spain

**Keywords:** Freedom of expression, Social vulnerability, Democracy, Western tradition, Human rights

## Abstract

Freedom of expression is a fundamental part of living in a free and open society and, above all, a basic need of every human being and a requirement to attain happiness. Its absence has relevant consequences, not only for individuals but also for the whole social community. This might explain why freedom of expression was, along with other freedoms (conscience and religion; thought, belief, opinion, including that of the press and other media of communication; peaceful assembly; and association), at the core of liberal constitutionalism, and constitutes, since the Second World War, an essential element of constitutional democracies. In a democracy, people should be allowed to express themselves to others freely. The paper, which is divided into five sections, points out that states are obliged to protect the exercise of that freedom not only because its very purpose is the common good and welfare of society but also because it is a requirement of any constitutional democracy. Otherwise, when people cannot express themselves, perhaps out of fear (not from ‘war’ but from different kinds of social pressure or ‘violence’ exerted by some lobbies, mass media, or governmental policies that are at odds with respect for the plurality of opinions), vulnerability arises. This weakens not only those individuals that are not allowed to express their thoughts but also those who do not dare to do it – or even not to think for themselves – under certain environmental pressures (exerted by states, international organizations, social media, or financial groups, lobbies, etc.). In the end, the decline of freedom of expression makes most people more vulnerable and jeopardizes the whole democratic system.

## Introduction

Freedom of expression is a fundamental part of living in a free and open society and, above all, a basic need of every human being. From a legal perspective, freedom of expression was, along with other freedoms (conscience and religion; thought, belief, opinion, including that of the press and other media of communication; peaceful assembly; and association), at the core of liberal constitutionalism. It also constitutes, since the Second World War, an essential element of constitutional democracies [1]. In the 1941 State of the Union address, on Monday, 6th January 1941, in proposing four fundamental freedoms that people ought to enjoy, Franklin D. Roosevelt stated that “[t]he first is the freedom of speech and expression everywhere in the world”, followed by other three freedoms (of worship, from want and fear). As America entered the war, these “four freedoms” symbolized America’s war aims and gave hope in the following years to a war-wearied people because they knew they were fighting for freedom. Roosevelt equated “Freedom from fear” mainly with overcoming war and violence. [2, 266–283].

This paper is divided into two parts. Part 1 will describe the relationship between public morality, freedom of expression and right to dissent in a democracy. This will be done with four sections: Sect. 2.1 will contain a brief presentation of the freedom of speech in the origins of modern constitutionalism; Sect. 2.2 will show the inextricable link between democracy and public morality and the two main models of the latter (libertarianism and perfectionism); Sect. 2.3. Argues that, since public morality is a constituent part of any democratic society and a deliberative democracy requires that decisions be the product of fair and reasonable discussion and debate among citizens, public morality should be also shaped by citizens through the exercise of freedom of expression. In doing so, I will describe how to combine, in an open and plural society, the private morality of individuals in the social realm and the public morality reflected in the legal realm, and how democratic systems should allow – and even foster – through the exercise of freedom of expression, a constant flux between private moralities and public morality; and Sect. 2.4. Argues that the freedom of expression necessarily requires the right to dissent because that is an essential part of its exercise, particularly in a deliberative democracy, and a human need according to the Aristotelian characterization of man as a political animal.

Part 2 will describe how the current freedom of expression crisis weakens society, making individuals less engaged in the community, more isolated, as if they were living among strangers, and, consequently, much more vulnerable. This part will be developed in four sections: Sect. 3.1. Will show how the freedom of expression constitutes a condition for political liberalism (enhancing human development and social happiness); Sect. 3.2. Will focus on the freedom of expression as a condition for democracy; Sect. 3.3. Will show the threads of freedom of expression today, analyzing the vulnerable effects of the cancel culture; and Sect. 3.4. Will show, as a particular case of criminalization of dissent, recent examples from Spanish law. Finally, some concluding remarks will be made.

## Public Morality, Freedom of Expression and Right to Dissent in a Democracy

### The Making of Freedom of Expression in Modern Constitutionalism

Freedom of expression is one of the most complex fundamental rights in modern Constitutions. This complexity is not new. It always has been [3]. Freedom of expression was already very present in the European enlightened cultural environment. Kant affirmed that “everyone has his inalienable rights, which he cannot give up even if he wishes to, and about which he is entitled to make his own judgments.” Furthermore, he added that among them was:

the right to publicly make his opinion known as what he considers unjust to the community in the sovereign’s provisions. To admit that the sovereign cannot even be mistaken or ignorant of anything would be to imagine him as a superhuman being endowed with heavenly inspiration. Consequently, the freedom of writing is the only defender of the rights of the people, as long as it remains within the limits of respect and love for the Constitution in which it lives, thanks to the liberal way of thinking of the subjects, which are also instilled by that Constitution, for which the writings further limit themselves mutually in order not to lose their freedom [4–6].

This cultural environment had consequences in the legal sphere [7]. First of all, in France, where on 26th August 1789, the Declaration of the Rights of Man and the Citizen was drafted, Article 11 of which reads as follows:

The unrestrained communication of thoughts and opinions being one of the most precious rights of man, every citizen may speak, write, and publish freely, provided he is responsible for the abuse of this liberty, in cases determined by law.

This principle was taken up two years later in the first French Constitution, 3rd September 1791, by establishing as natural and civil rights “the liberty to every man to speak, write, print, and publish his opinions.” This freedom did not only germinate in Europe but also in America. A few months after the aforementioned French constitutional text, the United States adopted the First Amendment to its 1791 Constitution:

Congress shall make no law respecting an establishment of religion, or prohibiting the free exercise thereof; or abridging the freedom of speech, or of the press, or the right of the people peaceably to assemble, and to petition the Government for a redress of grievances.

It is possible that the following statement by Alexis de Tocqueville emerged from the North-American experience:

Sovereignty of the people and freedom of the press are two entirely correlative things. Censorship and universal suffrage are, on the contrary, two things that contradict each other and that cannot exist together for long in the political institutions of the same people [21:3].

In Spain, freedom of expression emerged in the context of the War of Independence (1808–1814). Specifically, the Cortes of Cadiz approved two Decrees in this regard, one of 10th November 1810 and the other of 10th June 1813. Article 371 of the Constitution of Cadiz (1812), taking up Article I of the 1810 Decree, provided:

All Spaniards are free to write, print and publish their political ideas without the need for any license, revision or approval prior to publication, subject to the restrictions and liability established by law.

The 1869 Constitution was particularly relevant because it referred, for the first time, to the oral expression of thought, in its Article 17:

Nor may any Spaniard be deprived: Of the right to freely express their ideas and opinions, either orally or in writing, by means of the printing press or any other similar process.

Until then, freedom of (oral) expression was understood to be included within the freedom of written expression, that is, freedom of the press and printing press.

The other Spanish Constitutions took up freedom of expression in general from that time onwards, sometimes explicitly mentioning orality. Thus, for example, the Preliminary Title of the Draft Federal Constitution of the First Spanish Republic (1874) after stating that “Every person is assured in the Republic, without any power having the power to inhibit them, nor any law authority to diminish them, all natural rights”, included “the right to the free exercise of thought and the free expression of conscience.” Shortly afterward, the Constitution of 1876 established, in Article 13, that:

Every Spaniard has the right: To freely express their ideas and opinions, either by word or in writing, by means of the printing press or any other similar process, without being subject to prior censorship.

In similar terms, this right was enshrined in Article 34 of the Constitution of the Second Republic (1931):

Everyone has the right to freely express their ideas and opinions, using any means of dissemination, without being subject to prior censorship.

However, one might ask how the exercise of this right worked in practice throughout Spanish constitutionalism.^1^ The answer can be found in the literature showing the complexity of exercising this freedom [8]. During the Liberal Triennium (1820–1823), for example, it seems that the freedom of expression encountered two groups that threatened its exercise within the strict constitutional framework. First, there were some clergymen, whose sermons could become highly critical of the constitutional regime, thus leading to the approval of a statute (‘Orden’) of 30th April 1821. Second to object were some in the exalted sector of liberalism, which used freedom of the press to incite disobedience, slander, disorder, and anarchy. As a result of the concern for the correct exercise of freedom of expression, several Decrees were passed during the Liberal Triennium: 22nd October 1820, and the complementary Decrees of 17th April 1821 and 12th February 1822. The first Spanish Criminal Code also included several criminal offenses, which came to limit the framework for exercising this freedom. And so have all the criminal codes up to the present day (1848/50, 1870, 1928, 1932, 1944, and 1995).

This brief historical introduction to the freedom of expression is enough to show that the exercise of this right, present since the origins of modern constitutionalism, has always had – and will continue to have – enemies. In the nineteenth century, it was some ecclesiastics and some exalted liberals: some preventing expression on matters of faith and morals, others inciting disobedience, slander and disorder. At present, some essential requirements for the exercise of freedom of expression in the framework of plural and mature democratic societies are notably neglected: to reflect critically on the problems, to think for oneself, respectfully express one’s ideas, and adopt a positive attitude of listening to others, in order to learn from everyone and in particular from those who do not share one’s way of thinking. These are the inescapable conditions of freedom of expression that the law must safeguard and foster. Otherwise, the free development of each individual’s personality is precluded (art. 10 CE), other freedoms are curtailed (such as those of thought and conscience), and democracy becomes a formal or aesthetic reality, void of content and subject to various forces of totalitarian domination. In this vein – as will be seen –, to prohibit the expression of dissent on controversial issues (such as the beginning and end of life, family, sexual morality, etc.) would be to return – at the very least – to the regime of freedom of expression of the early nineteenth century, in which questions of Christian faith and morals were excluded from the exercise of freedom of expression, and anyone who expressed dissent was punished.

I can understand – although one might disagree with it – that this would happen in the framework of a confessional or denominational state (as in the nineteenth century). However, it would make no sense in today’s framework of constitutional democracy. In this sense, the use of double standards when judging – or even legislating – the scope and limits of the exercise of freedom of expression and freedom of information, depending on for what and for whom, preventing some from even speaking and allowing others to insult and slander, not only constitutes an unequivocal sign of a broken and sick democracy – perhaps deathly – but it leaves all its individuals, both those who choose to conform to the majority opinion and those who are willing to express their disagreement, in a vulnerable situation. This vulnerability of individuals is a reflection of the fragility of democracy. The strengthening of democracy also implies strengthening individuals and vice versa. For this, the safeguarding and fostering of freedom of expression constitute a *sine qua non* requirement.

### Public Morality and its Models: Libertarianism vs. Perfectionism

In a true, rather than in a merely formal democracy, the exercise of freedom of expression by citizens should be the fundamental element in shaping the public morality of society. A citizen might feel more or less identified with a particular public ethic, but he/she should never ignore it. Furthermore, the law should promote the contribution of each individual in the never-ending process of shaping public morality. However, what exactly is public morality?

Public morality is a reality, whether we like it or not [4, 9–12]. Ultimately, it is that set of beliefs and values generally assumed by society, by each society, which has its differential elements depending on the geographical context and that tend to change and evolve over time [13, 268–277]. The values that underpin a country’s society at a given moment in history – like the transition, for instance – can have little to do with the values that the same society is underpinned by sometime later.^2^

A society cannot fail to be based on principles and values that its citizens widely accept. The common acceptance of these principles and values (public ethics) builds stable societies, even though some people may claim otherwise or proclaim themselves liberal and present themselves as supposedly neutral towards any value or ethical principle. Being liberal does not imply being unethical, but it entails assuming a specific type of ethics. There is a liberal who tends to present himself as tolerant and boasts of respecting all positions but accuses those who do not share his stance of being intolerant and of attempting to impose their ethics on the rest of society. This is a well-known demagogic device that nevertheless leaves many without knowing what to say or how to respond. This is the stance adopted by the so-called ‘Libertarianism’ movement in the United States, whose supporters take the liberty of criticizing – and even disqualifying – those who maintain that society needs somewhat more demanding ethical rules that guarantee a minimum of standards of justice that are indispensable for stable and peaceful coexistence [14].^3^

For libertarians, freedom is the fundamental ethical principle. Invoking an ethical demand to reduce a person’s capacity to make choices and decisions in any area is interpreted as an illegitimate interference or an unacceptable encroachment. Libertarians understand that no one can be constrained or limited in their decisions by any ethical imperative that seeks to impose on one’s own will [3, 6, 13, 15–24].^4^ For this conception, it would be just as unacceptable to force someone to end their life as it would be to prevent someone from being able to make that decision if that is what they desire. It would be just as unacceptable to force a woman to have an abortion as it would be to forbid her from having one if that is what she wishes. It would be just as unacceptable to force someone into prostitution as it would be to stop them from doing so if that is what they want. It would be just as unacceptable to force someone to try drugs as it would be to prohibit them from doing so if this was their will, and so on. The same applies to many other areas like economics (capitalism, communism, liberalism, neoliberalism, protectionism, etc.) or sexuality (prostitution, pornography, bigamy, pedophilia, polygamy/polyandry, polyamory, incest, etc.), among others.

At the other extreme is the so-called ‘Perfectionism’ trend, which upholds more rigorous standards of public ethics. Its supporters believe that it is the virtue that should guarantee justice and social peace and that the climate of the political community should favor the virtuous conduct of its individuals. In short, they argue that society should contribute to shaping citizens of exemplary conduct, thus positively impacting society as a whole. According to this perspective, the fundamental ethical principle is not so much the freedom of choice advocated by ‘libertarianism’ but the idea of the good, the promotion of virtuous conduct, and the idea of the good shared by society. From this point of view, in order to tackle complex issues (euthanasia, abortion, drugs, prostitution, incest, polygamy, etc.) [12, 521–538, 21, 320–358], the important thing would not be to “let everyone do what they want” – because the political community should neither oblige nor prohibit (libertarians) [25]^5^ – but to establish “what is good for the individual and society as a whole,” a doctrine which has its roots in authors such as Plato [26], Aristotle [27], and Thomas Aquinas [28, 29], among others [2, 7, 17, 22–27, 30–58].^6^

Both currents, present in some form in all Western societies, are irreconcilable and struggle to impose themselves on the public ethics of each political community. To attain this, they need to introduce their presuppositions into education, culture, the media, social networks, cinema, literature, etc. Moreover, the quickest way to achieve this is to introduce them into government programs and to use the law as a tool for change. With the law, it is somehow swift and easy to reform education, making it possible to mold the minds of an entire generation in little more than a decade through the curricula of compulsory education. By changing the laws [2, 7, 9, 25–27, 30–50, 57], history and language can be changed, even at the cost of trampling its scientific status with ideological or partisan manipulations. Power can favor a particular type of culture (cinema, art, etc.) by subsidizing television channels and media groups that disseminate it and ignoring others; it can also bail out certain companies while leaving others to go under, etc.

This is well known to everyone, but it is occasionally forgotten that the libertarian currents are not as innocent as they preach and use the law to impose their principles, as much or more than the perfectionist currents, despite presenting their measures or legal reforms as ethically aseptic. In this sense, promoting a law that prohibits euthanasia, opting instead to promote palliative medicine and care for the terminally ill, so that they prefer to live rather than die, should be considered as moral – or immoral, depending on one’s position – as promoting another law which financially supports those who decide to end their lives [59, 60]. One can disagree about what is ethically correct, but it cannot be stated that the first of these proposals imposes an ethical option and the other does not. There is a clear and undeniable ethical background in both [57], even if the second neither prohibits nor compels and subsidizes an individual’s wish to die, while the other prohibits killing and funds better care for the sick by facilitating their access to palliative care in the hope that they will prefer to continue living.

In my case, I must admit that, precisely because I believe in and love freedom, I do not identify myself with libertarians or perfectionists. I do not identify with the former because it does not seem reasonable to me to maintain that freedom, understood as a mere capacity to choose, is a guarantee of a truly human life and society; in fact, it is evident that there are decisions that make one better as a person (for example, trying to work well and in a spirit of service to others) and others that make one worse (working shoddily, trying to look good or cheating others). Nor do I identify with the latter when perfectionism is interpreted as the imposition of an idea of the good that is totalitarian and disrespectful to the individual because I understand that it should be each person who freely decides for the good and should never adhere to it “forced” by a paternalistic government or laws that do not allow one to choose the opposite.

I understand that the law must safeguard and foster minimum requirements of justice, reflected in the three basic rules of the jurist Ulpian: live honestly, do not harm others, and give each one what is theirs. However, the State and the Law should not go beyond these requirements (because their task is not to make citizens good but to create the minimum conditions of justice that allow a stable and peaceful coexistence). In reality, only everyone can become good (and not just fair) when they freely choose to do what is good (not merely fair) and do it for a good or right reason (and not because it is so legally prescribed). Neither the State nor the Law can make me good: I can only become good when I freely choose to do what is good and do it for a good (or right) reason. In case the State or the Law forced someone to do good (in the event that this was legally prescribed), that by itself would not make him/her a good person because there would be a lack of freedom (to do it for being good and not for being legally mandated). Only from freedom – not from imposition or coercion – can one become good by doing good (although it is also possible to adjust one’s conduct under the law, not because it is legally prescribed but because it understands and identifies with the good that the legal norm pursues).

### Public Morality, Deliberative Democracy and Freedom of Expression

The fundamental question concerning public morality is not whether it is possible or desirable for a society to have it or not to have it. In reality, society always has it and can never cease to have it. What is relevant, especially in a democratic society, is how and who should shape the values and principles that govern that society. In my view, the principal shapers of public ethics should be the citizens themselves. I think that in a free and plural democracy, the State should not be the primary agent shaping the fundamental values that underpin social coexistence, nor should the big business, media and financial groups. That is a fundamental requirement of deliberative democracy [29, 39, 40, 61–68].^7^ Otherwise, democracy becomes corrupted and turns into demagogy, quickly leading to an authoritarian or totalitarian regime. This process of democratic corruption is precluded when the political freedom of a community is based on the sum of individual freedoms, not in the abstract, but in their concrete and free exercise.

For that reason, citizens need to think for themselves, express their thoughts publicly in a climate of freedom – regardless of what they think – and contribute, within their means, to shaping the public ethics of the society in which they live. Furthermore, in a democracy, public ethics should be a dynamic reality, in constant movement, even when some of its parts have crystallized or have been enshrined in a legal norm. Therefore, the law should not prevent citizens from being able to think and express doctrines that are contrary to the hegemonic public ethics at a particular time. Hence, the importance of freedom of expression is that, although it is not the most important fundamental right (the right to life, for example, is the first and makes the exercise of the others possible), it is the most fundamental and genuine right in any democracy.

One could ask oneself the following questions: how can the dilemma between the exercise of individual freedom (in accordance with the personal ethics of the citizen) and the general character of the law (reflecting public ethics) be resolved? How can different ethics coexist in the same society, namely public ethics (principles more or less common to the majority and endorsed by law) and different private ethics (of each citizen)? To what extent, in a democratic regime can the State prohibit dissent or prevent a citizen from expressing their private morality when it is contrary to or different from public morality? This is, undoubtedly, a key question in any democracy worthy of the name. On the one hand, it is logical and understandable that the State should enact laws that reflect the prevailing public ethics of society at a given time. To do otherwise would be suspicious or worrying. Once the law has sanctioned some principle of public ethics, it is reasonable to prohibit conduct that violates it. However – and here comes the critical nuance – it is one thing to prohibit conduct contrary to fundamental values and quite another to prohibit opinion. The law should never prohibit the expression of dissenting opinions, as long as they do not constitute a severe and real threat to coexistence (encouraging hatred, violence, etc.) or a direct attack on the rights of third parties.

It should prohibit, however – in my opinion – dissenting expressions which have the effect of excluding, mocking and humiliating those who do not share any of the principles of a specific public morality should not be admissible, even if the law allows it in some cases. Let me put an example. If public morality, reflected in legislation, did not allow people to go into the street naked, they could be sanctioned, but a constitutional democracy should never punish those who, judging it a good thing to be able to go naked in the street – even if they were not legally allowed to do so – could at least express their opinion and defend – without the threat of any punishment – their dissenting stance; that is to say, support – for the free development of one’s own personality – the desirability of the citizen being able to go naked in the street or propose the creation of zones or urbanizations in which people could go naked in the street. The State should not deprive that person of the freedom to express what they think. In this fashion, freedom of expression would play its proper role in bridging the gap between ‘public morality’ and ‘private morality,’ promoting a constant ebb and flow between one morality and the other. This dynamism, characteristic of a genuinely free and pluralistic democracy, would prevent the totalitarian attitude of those who demand maximum freedom of expression when they claim their ideas for a new ‘public morality’ (think of May 68) and prohibit dissent when they have already succeeded in shaping a public morality following their ideas (which is what is currently happening with legislation on sexual freedom and gender identity) [6, 13, 19, 19–24, 52–54, 57, 59, 67, 69, 70–74].

Continuing with the previous example, if the day arrives in which nudism – being able to wander around the street naked – becomes part of public morality, should the State be allowed to prohibit the expression of dissent? Absolutely not; it should not be prohibited at all, and even less so – as is done with certain groups – by resorting to the principle of non-discriminatory freedom, with a line of argument as simplistic as the following: “if everyone can walk down the street as they wish, why discriminate against the nudist group? If you are not forced to go out naked, why do you seek to impose your position on everyone, preventing anyone from being able to go out naked? Why don’t you let others make their own moral choices?”. If one admits that the fundamental source and criterion of the principle of non-discriminatory freedom is of a strictly subjective order and does not take into account the good of the community as a whole – because one believes that this good does not exist, that the good is always something private, subjective and immanent –, the egalitarian or non-discriminatory argument can become, in the hands of the State, a dangerous tool of totalitarian imposition, which is incompatible with an authentic constitutional democracy.

The tendency to excessively restrict or entirely prohibit freedom of expression when talking about specific groups is another sign of the current fragility of exercising this fundamental right. Some argue that the mere dissenting expression about the ways of life of particular groups would constitute an incitement to hatred that, as such, should be criminalized. Although it may seem exaggerated, for them, it is not at all because they understand this discrepancy concerning some forms of life as an affront or offense to the group of people who shape their lives according to that model. As it is not possible to generalize this principle in all cases (because, if done, one could not disagree on almost anything), it starts from the ‘victimization’ of a group (based on the commission of civil and criminal offenses by some against those who belong to the group) to extend the criminalization of any discrepancy with respect to that group because it is considered hate speech. They lose sight, however, that this type of criminalization usually has a boomerang effect, which goes from one extreme to another, and rarely settles in the reasonable point of moderation that would imply punishing only those who inflict insults, humiliations or injuries, and not criminally prosecute the rest of society for expressing their views on any model of life, as happens with the discrepancy towards other groups, some of them much reviled over many years or even centuries. Of course, it seems more reasonable to punish those who insult and commit aggressions against a person (regardless of the group to which they belong) and to allow citizens to express their ideas about any way of life (whether or not they refer to a group of any kind – religious, professional, cultural, sexual, etc.) [69, 299–321].

Another symptom that demonstrates the poor quality or maturity of a democracy is the frequent use of labels or clichéd expressions to disqualify those who disagree (fascist, communist, fanatic, nationalist, pro-independence, philo-ethnic, homophobic, male chauvinist, far-right, far-left, etc.) This recourse, which is so frequent, especially in politics and the media, and which usually implies contempt or gross simplification of reality, does not seem to be the best way to promote freedom of expression and the spirit of dialogue that should characterize a constitutional democracy. Nevertheless, the problem for some is that they consider themselves so clear-headed and so entrenched in their ideological positions that they are unwilling to accept that, through dialogue and plural debate, consensus can be reached that is far removed from or alien to ‘their’ truth. When the majority supports their principles (they become public ethics), they prevent and prohibit dissent with the coercive force of laws and media pressure. However, when the majority does not share ‘their’ truth (private ethics), they promote disagreement in the name of freedom of expression and the right of minorities against the supposedly illegitimate impositions of the majority.

In summary, I argue that public morality should not be the result of the will of the State, nor of powerful lobbies (politicians, business people, media, and financial egalitarians), but the result of the exercise of the freedom of each and every citizen, who is called upon, to the extent of their possibilities, to shape the public ethics of their political community. In correspondence with what I have just stated, esteemed reader, I do not intend to convince you of anything, much less to convince you to think as I do. My purpose has been to freely express my critical reflections on a vital issue in any democratic society, and in ours in particular, in the hope of helping you to think for yourself and to encourage you to also have the courage to contribute, through your free and active participation, to the flourishing of a freer, more open, plural and mature democracy.

### Democracy, Freedom of Expression and Right to Dissent

There is no democracy without freedom of expression, and this is only real if there is room for different, minority, or dissenting thoughts. Since this is the essence of democracy, dissent is essential in a democracy [23, 848–852]. Rejecting dissent would lead to the end of deliberative democracy [20, 75]. “Rule of majority is an integral part of democracy, but majoritarianism is the antithesis of democracy” [76].

Nobody would deny that dialogue and tolerance are critical to a pluralistic and inclusive democracy. However, few see dissent in a positive light, and even fewer are willing to accept it and engage in dialogue with it. Today’s culture is based on the idea that “the enemy is the other, the stranger” (Meinecke), that “hell is the other” whose gaze and judgment limit me, expose my limitation, humiliate me, not being able to escape from that judgment of others in the knowledge of myself (Sartre) [77]. Hence, the other can – and perhaps, must – be endured if their ideas and opinions are identical or similar to mine. If they are different but at least able to remain silent, their presence in society is still bearable and tolerable. Nonetheless, if one dares to disagree, to give reasons that may contribute to public deliberation, they should be silenced immediately. The dissenters are those who cross this line and dare to express their opinion publicly (alien or contrary to the majority), and this makes them a *persona non grata* and an enemy, thereby acquiring a new social – and, in part, also legal – vulnerable status because their rights happen to be more those of a law of war than those of a state governed by the rule of law.

It is paradoxical that today’s culture, so diverse and inclusive in theory, is hardly so in reality with relation to dissenting opinion. A US journalist based in Poland, Anne Applebaum, has just testified to this in a recently published book that has become a best-seller [36]. Applebaum experienced the consequences of expressing one’s own ideas, both in politics and society, to the point of being abandoned by educated people she had considered good friends. She also recounts how Western democracies are being besieged by authoritarianism that penetrates society with simple, false – or half-false – and radical messages, but which are attractive and have an effect. But reality is not reducible to simple messages, and simplistic approaches often contain falsehoods or half-truths on which the authoritarian mentality feeds. Political psychologist Karen Stenner argues that those who want to impose their own way of seeing reality do not tolerate complexity, nor do they wish to understand that certain events are rooted in a variety of factors [36].

Dissent is seen as something annoying and unpleasant which is to be stoically endured, but not as an essential means of enriching one’s own thinking, let alone as a requirement for public deliberation of what is suitable for each society. Hence the title of Arthur C. Brooks’ book: *Love Your Enemies: How Decent People Can Save America from our Culture of Contempt* [42]. For Brooks, society will be saved by those who can love their enemies, not by those who indulge in a culture of contempt for their enemies, i.e., those who disagree, those who think differently.

Disagreement is required for one reason of elementary education and for another one of common sense in having to coexist with people with different visions in the framework of a plural democracy. However, there is another more important reason: only disagreement allows us to reach a broader and more complete vision of reality, which is never simple, flat, and uniform, but rich, complex and multifaceted. The scientist Karl R. Popper said that “the increase of knowledge depends entirely on the existence of disagreement.” It has also been said, and rightly so, that “the ability to listen to intelligent people who disagree with you is a hard talent to find” (Ken Follet). It is easier to cuddle up to those who please us, as children do, because, as Kant said, “it is so easy to be a minor!” The opposite happens to me: I am attracted to those who have the courage to disagree. The same happened with the French philosopher Michel de Montaigne, who said that “when they contradict me, they arouse my attention, not my anger; I offer myself to those who contradict me, who instruct me. The cause of truth should be the common cause of one and the other”. A society is more mature and democratic when its individuals can be friends with those who do not think as they do, see those who disagree with their ideas as someone who helps and enriches them, and not as a nuisance and an obstacle to their fulfillment. To be friends only with those whose ideas we like and share is to remain immature, to renounce a fullness that implies the recognition that one does not have the whole truth and that I can only get closer to it by listening to and understanding the point of view of others.

There are those who understand democracy as the source and oracle of truth and goodness. They conceive the State as the modern inquisition whose function is to decide what can and cannot be said, what can and cannot be disagreed about. They are entirely wrong. The essence of democracy lies in guaranteeing fundamental freedoms. The first one in the public sphere is to allow everyone, without excluding anyone, to contribute to public deliberation. Freedom of expression is only real if it includes the right to disagree on any topic, without exception, because this is the essence of democracy in a state governed by the rule of law, not the defense of certain goods or truths to the point of prohibiting dissent. Moreover, “democracy is strengthened by disagreement. Unanimities are the path to totalitarianism,” as stated by the Argentinean politician Ricardo Balbín. What truly threatens democracy is not a way of understanding the good or truth but rather prohibiting – legally, politically, mediatically or socially – dissent, imposing a “culture of cancellation” that leaves the civil, professional and media dissenter dead, euphemistically ‘cancelled’, as has been lucidly described by Alan Dershowtiz [49].

Hence, I totally agree with Voltaire’s apocryphal saying: “I do not share what you say, but I will defend to the death your right to say it” [78]. This should be the spirit and mentality of a genuinely democratic society. Otherwise, each time one is prevented from dissenting or, in doing so, is sanctioned, vilified, insulted, stigmatized, or labeled (as “fascist,” “communist,” “homophobic,” “populist,” etc.) by others, particularly in the political, media or academic sphere, we are becoming a less pluralistic and democratic society, and a more authoritarian or totalitarian one.

## The Decline of Freedom of Expression and the Increase of Social Vulnerability

### Freedom of Expression as a Condition for Political Liberalism: Human Development and Social Happiness

As suggested at the beginning of this article, freedom of expression is not only a fundamental part of living in a free and open society, but also – above all –, a basic need of every human being. In this vein, early modern constitutions equated freedom with happiness. More specifically, the *Virginia Declaration of Rights* linked “the natural right to pursuit of happiness” to security, life, liberty and property [79, 318]. Section 1 (‘Equality and rights of men’) of Art. I (‘Bill of Rights’) reads as follows:

That all men are by nature equally free and independent and have certain inherent rights, of which, when they enter into a state of society, they cannot, by any compact, deprive or divest their posterity; namely, the enjoyment of life and liberty, with the means of acquiring and possessing property, and pursuing and obtaining happiness and safety [79, 318].

George Mason’s expression “pursuit of happiness” highly influenced Thomas Jefferson when drafting the text of the *American Declaration of Independence*, whose Prologue reads:

The unanimous Declaration of the thirteen United States of America (…).

We hold these truths to be self-evident, that all men are created equal, that they are endowed by their Creator with certain unalienable Rights, that among these are Life, Liberty and the pursuit of Happiness. That to secure these rights, Governments are instituted among Men, deriving their just powers from the consent of the governed…”[33].

Some years later, the *Declaration of the Rights of Man and the Citizen* also referred to the connection between constitutions and happiness of all:

…in order that the grievances of the citizens, based hereafter upon simple and incontestable principles, shall tend to the maintenance of the constitution and redound to the happiness of all” [48].

The link between happiness and freedom was explicitly stated in some European constitutions. The French 1793 Constitution was preceded by the mentioned *Declaration*, Prologue of which stated the following:

“The French people, convinced that the forgetfulness of and contempt for the natural rights of man are the sole causes of the misfortunes of the world, have resolved to set forth these sacred and inalienable rights in a solemn declaration, in order that all citizens, being able constantly to compare the acts of the government with the aim of every social institution, may never permit themselves to be oppressed and degraded by tyranny, in order that the people may always have before their eyes the bases of their liberty and their happiness, the magistrate the guide to his duties, the legislator the object of his mission. Accordingly, in the presence of the Supreme Being, they proclaim the following declaration of the rights of man and citizen:

1. The aim of society is the general welfare. Government is instituted to guarantee man the enjoyment of his natural and inalienable rights. (…)” [47].

Freedom of expression was amongst the first and most important fundamental rights in modern constitutions. The French 1791 Constitution, in its Title I (‘Fundamental Provisions guaranteed by the Constitution’), secured it among other natural and civil rights:

“Liberty to every man to come and go without being subject to arrest or detention, except according to the forms determined by the Constitution;

*Liberty to every man to speak, write, print, and publish his opinions without having his writings subject to any censorship or inspection before their publication*, and to worship as he pleases; (…)” [80].

Mill’s work *On liberty* regarded freedom of expression as one of the most fundamental freedoms [81]. He presented “perhaps the most famous liberal defense of free speech” [64]. In the first footnote of chapter II, he made the following strong statement:

If the arguments of the present chapter are of any validity, there ought to exist the fullest liberty of professing and discussing, as a matter of ethical conviction, any doctrine, however immoral it may be considered [42, 81].

He argued that everyone should be allowed to give his/her opinion:

If all mankind minus one were of one opinion, and only one person were of the contrary opinion, mankind would be no more justified in silencing that one person than he, if he had the power, would be justified in silencing mankind [43, 81].

For Mill, freedom of expression was necessary for the dignity of persons. The price of curtailing it to get “a sort of intellectual pacification” implied to sacrifice “the entire moral courage of the human mind” [51, 81]. He thought that speech should be protected because it is the path that leads to truth: if we suppress an opinion, it may turn out to be true. To assume otherwise is to assume that we are infallible, which is not the case [1–15, 15–28, 30–60, 69–74, 76–79, 81, 81–102].^8^ In his view, “if we ban speech the silenced opinion may be true, or contain a portion of the truth, and that unchallenged opinions become mere prejudices and dead dogmas that are inherited rather than adopted.” Mill maintained that “free speech fosters authenticity, genius, creativity, individuality and human flourishing” [64].

Mill maintained that the big loser of censorship was not the one who was not allowed to express his/her opinion, but humanity in general and his/her society in particular:

Were an opinion a personal possession of no value except to the owner; if to be obstructed in the enjoyment of it were simply a private injury, it would make some difference whether the injury was inflicted only on a few persons or on many. But the peculiar evil of silencing the expression of an opinion is, that it is robbing the human race; posterity as well as the existing generation; those who dissent from the opinion, still more than those who hold it [41, 42, 90].^9^

Hence, the limitation on freedom of speech should be based upon “one very simple principle,” the so-called harm principle:

…the only purpose for which power can be rightfully exercised over any member of a civilized community, against his will, is to prevent harm to others [37, 81].

As seen, freedom of expression was also among the four main freedoms in the famous Roosevelt speech in 1940, when America entered the Second World War.

### Freedom of Expression as a Condition for Democracy

In the context of Western democracies, free speech became even more relevant, since each citizen should have his/her say and be allowed to contribute in shaping his/her political community. In authoritarian and totalitarian regimes, in both the past and the present, freedom of speech is radically curtailed, particularly the voice of those who do not agree with the political regime, its laws, as well as the public morality they reflect. As seen above, in democratic states, public morality is supposed to be the result of private moralities freely expressed by individuals. Citizens, by thinking and expressing their views, contribute to shaping public morality in their social and political community. Moreover – as we saw – in a real democracy, public morality is somehow permanently on the move because there is a constant flux between private moralities and public morality. Freedom of expression makes this flux possible. The necessary requirements are that citizens think for themselves and have the courage to express their views. Such requirements are not easy, but laws should promote and protect both things and, particularly, the freedom of expression. Otherwise, democratic societies suffer and individuals become “more vulnerable”. What do I mean by becoming “more vulnerable”?

The ability to reason and communicate your thoughts, views or feelings are two fundamental traits of human beings. The school of Salamanca (Francisco de Vitoria and Domingo de Soto, among others) defended Native Americans because of their humanity, by arguing that their dignity should be respected as such because of their ability to reason and communicate. Hence colonizers had the right to spread the Gospel (without implying the right to become the owners of natives’ land) because everybody has the right to communicate and express what they think. In other words, communication is a human need that derives from the inherent social dimension of the human being. Depriving someone from expressing what he/she thinks means to undermine him/her as human being or to treat him/her below his/her human dignity.

The connection between freedom of expression and human development was also explicitly stated by the ECtHR in *Handyside v. United Kingdom*:

Freedom of expression constitutes one of the essential foundations of [a democratic] society, one of the basic conditions for its progress and for the development of every man [5].^10^

### The Threats of Freedom of Expression Today: Cancel Culture and Vulnerability

When the social environment and state laws do not allow individuals to express their own ideas because they are considered wrong or harmful to the whole society, to a vulnerable group of individuals o to someone in particular, two important things happen:

1. The above-mentioned constant flux between private and public morality is broken, so some individuals or part of the society are not allowed to communicate or express their views, undermining two fundamental traits of democracy: plurality and inclusiveness;
2. The common or inherent vulnerability of such individuals increases, among other reasons because, not being allowed to shape their own political community by expressing their views, they somehow become second-rate citizens.

The idea of vulnerability is then at the fore front of the exercise of freedom of expression [83]. Everybody agrees that the freedom of expression of vulnerable and marginalized people – including minorities – should be defended and even empowered [33, 63].^11^ However, protecting and empowering vulnerable people by conditioning, restricting or punishing the free expression of ideas of those who do not belong to vulnerable groups, in a way not adequately justified and “proportionate to the legitimate aim pursued,” [33, 63]. could cause the vulnerability of the majority for being deprived of expressing their views. Moreover, such disproportionate restrictions would not help to enhance a culture of respect and tolerance towards the most vulnerable or/and marginalized people.

Hate speech has its role, but sometimes it is disproportionately used as a tool to restrict the freedom of expression of a person belonging to a dominant majority group towards of a person belonging to a vulnerable group [38, 51, 83],^12^ particularly when some of them are supported by powerful lobbies that exert a remarkable influence over some international organizations, some global companies, and most of the mass media and film industry (e.g. Hollywood).

Democratic states need to promote free and strong social environments in which the empowerment of the freedom of expression of vulnerable and marginalized groups does not bring along with it the vulnerability of the rest or, in other words, that the defense of minority rights is not done at the expense of curtailing or prohibiting the expression of the rest of the society. In this vein, a free and democratic society should be characterized by its capacity not only to allow the expression of private moralities that are against the public morality reflected in the enforceable current laws, but also to welcome dissenting opinions on controversial issues and the openness towards pluralism and diversity. This does not seem to be the prevailing cultural environment of Western democratic societies, in which many people are afraid of expressing their views; these are societies in which two languages are spoken, one in the private and the other in the public arena, in which political correctness dictates what should or should not be said, punishing with the social or civic death those who dare to think for themselves and express their thoughts. It is what has been called, as seen above, cancel culture.

Cancel culture leaves individuals who dare to express views that are regarded as heterodox in a state of vulnerability. Cancel culture consists precisely in taking away support for individuals, their careers, popularity and/or fame because something they expressed – by words or deeds – is considered unacceptable. “To be ‘cancelled’ is effectively to be boycotted, with the intent that the person will be ostracised and no longer benefit financially, personally or professionally from their elevated position” [66].^13^

One might think that this mainly affects public figures (Jimmy Carr, J.K Rowling, Chris Noth, Chrissy Teigen, etc.) [66], but it might also touch upon common people from different social and professional backgrounds, particularly in politics, public administration – particularly in the judiciary –, journalism, academia and in other cultural environments, in which experiences of surveillance, threats and harassment have already produced a chilling effect on the free exercise of expression [1, 227–242, 228].^14^ In this regard, it has been shown how “[US] writers are not only overwhelmingly worried about government surveillance, but are engaging in self-censorship as a result” [62]. In fact – as a PEN report points out –, the assumption that writers “are under surveillance is harming freedom of expression by prompting writers to self-censor their work in multiple ways, including: a) reluctance to write or speak about certain subjects; b) reluctance to pursue research about certain subjects; and c) reluctance to communicate with sources, or with friends abroad, for fear that they will endanger their counterparts by doing so.” [1, 227–242, 228].^15^

In the European Commission’s first Rule of Law Report – and connected country chapters – published on 30 September 2020, “the concept of chilling effect was mentioned 20 times in relation to legal measures, political attacks, smear campaigns, abusive lawsuits and threats targeting journalists, civil society, judges and prosecutors” [33, 94, 103].^16^

Internet contributes to increase the perception of being under surveillance [33, 94, 103],^17^ but also of being more easily exposed to harassment, bullying or to different kinds of exploitation, particularly those most vulnerable [53]. In fact, cancel culture might, in the online environment, be even more severe and have a global impact. The exercise of freedom of expression in the digital world might make people even more vulnerable, leaving – as a Report for the United Nations Commission on Human Rights asserted – their voices “ignored and consequently left out of any debate” [61].^18^

The connection between freedom of expression, internet and vulnerability is undeniable [55, 56]. In this vein, the risk of being cancelled or excluded from debate when exerting freedom of expression in the digital world is high. Moreover, the dangers and threads that internet poses to vulnerable people have been detected and denounced by some European jurisdictions. In England, for example, a report of the Communications and Digital Committee, published on 22 July 2021 [99], warns how “vulnerable adults are often ignored in the debate around digital citizenship education.” In defining what a “vulnerable person” is, the report refers to “someone who is unable to look after themselves, protect themselves from harm or exploitation or are unable to report abuse.”^19^ The report recognizes that “vulnerable person” covers a wide range of people, but does not emphasize that on the Internet everybody, to some extent, becomes more vulnerable (and not just “those with physical disabilities or illnesses, neurodiverse individuals, care leavers, people with mental health difficulties, those with addictions, and homeless people.”). In fact, many common people have suffered, on the Internet, “harassment, bullying, exposure to harmful content, sexual grooming, exploitation, encouragement of self-harm, and access to dangerous individuals or information.”^20^ So there is indeed an urgent need to create a safe online experience: “an inclusive online environment is therefore especially important for vulnerable adults to ensure they can express their views freely online.”^21^ It is true that online “[a]nonymity can allow individuals, including those in vulnerable positions, to express themselves more freely and to challenge orthodoxies,” but “it can also embolden people to abuse others,”^22^ making them all the more vulnerable [91, 159–173].^23^

Norway has also paid attention to the risk of exerting the freedom of expression on the Internet from the vulnerability´s perspective. In February 14, 2020, a Freedom of Expression Commission was appointed with the assignment to “consider measures to promote an open, informed public discourse, including: (…) [m]easures to promote wide participation in the public exchange of ideas. In this regard, the Commission should, for instance, problematise the distinction between offensive statements that are not protected by freedom of expression and statements that are protected but which may nonetheless be perceived as challenging because they lessen vulnerable groups’ real opportunities to express views freely and participation in democratic processes.”^24^ A year later, the Norwegian Ministry of Foreign Affairs launched its international strategy for promoting freedom of speech in foreign and development policy [104]. The document recognizes that “freedom of expression and media freedom are under severe pressure” and that “[m]any countries, including a number of democratic countries, have introduced new restrictions that limit freedom of expression.”^25^ And further on, it touches upon one of the most delicate issues:

Legislation and mechanisms that are intended to provide protection against harmful and illegal speech must be developed in a way that safeguards the most vulnerable groups but does not lead to disproportionate restrictions on freedom of expression and information.”^26^

The document recommends a “Safe environment for freedom of expression,” and warns about “Online threats” as follows:

Freedom of expression means that all people have the right to express themselves freely in the public domain without fear of surveillance, censorship, discrimination, intimidation, or other forms of abuse. *Many individuals and groups lack both the opportunity and a safe environment to be able to participate in the free exchange of views, and there is a particular need to protect their right to freedom of expression*.^27^

Fostering a safe environment for freedom of expression means to safeguard the free exchange of opinions of all people, above all the vulnerable, and particularly those who are excluded from debate for daring to challenge orthodoxies, those who are boycotted or ostracized, or suffering a kind of social and civic death for expressing their views.

Some scholars in the field of social sciences denounce that some social engineering of language has poisoned Western democracies. In their view, such engineering of language advocates “censorship to protect the rights of marginalised and vulnerable groups, while paradoxically censors the right to expression of thought and infringing on a basic right of freedom of speech” [68, 175–201]. They argue that “political correctness is charged with giving carte blanche to the use of emotionally charged accusations (e.g., racist, sexist, homophobic) toward views that dissent from a supposed superior moralistic perspective. Political correctness ultimately complicates engagement between people who differ; rendering interactions and discourse shallow or uncomfortable” [68, 85: 443–445].

In addition to political correctness, another obstacle linked to vulnerability needs to be overcome to exert freedom of expression. Among US university students a peculiar kind of emotional vulnerability is growing: American undergraduates have become increasingly prone to a syndrome of “vindictive protectiveness”, whereby individuals attack anyone or anything that threatens their emotional wellbeing. Political correctness, and its various campus manifestations such as “safe spaces”, become a kind of pathology that not only harms the sufferer, but damages the capacity to argue and reason [45]. This tendency has been found in the US, very much in the terms Greg Lukianoff and Jonathan Haidt described in 2015 [22]. They argue that in the name of emotional well-being, college students are increasingly demanding protection from words and ideas they don’t like. In their view, this is disastrous for education—and mental health. In their view, “the very idea of helping people with anxiety disorders avoid the things they fear is misguided” [22].^28^ The same tendency can be seen in England and other European jurisdictions [8, 12, 14, 58, 60, 74, 75, 77, 81, 90–102, 105].^29^

People are becoming less tolerant towards dissenting opinions and views that are contrary to their own way of thinking or living, so they experience emotional distress and anxiety disorders. They feel comfortable in the digital world, in which everything is explained, seen, experienced and felt according to their own whims, and become unable to manage their feelings when differences and dissensions arise outside of their (digital) worlds. They take such dissenting opinions as a thread rather than a richness of social coexistence within plural and inclusive democracies. They overlook that “when people replace their need to defend themselves with a desire to learn, the possibilities for constructive cross-cultural interactions increase enormously” [82].

Postmodern society does not accept limitations, its individuals do not cope with them either, so any dissenting view is perceived as a threat, not as opportunity to learn, to improve or enrich oneself:

Learning requires people to acknowledge their limitations and to suspend their need to be right or to prove their competence. In so doing, they make themselves *vulnerable to others’ judgments* so that they can perform their jobs more effectively [82].

Here the expression *vulnerable to others’ judgment* is used in the sense of being open to criticism or to improvement. This is only possible in a social environment of safety, whereby that “people are well-intentioned and (…) that well-intentioned actions will not lead to punishment” is assumed. In a few words, “people (…) need to feel safe” [82].^30^ But this is not a trait of our postmodern society, in which self-righteousness unfortunately leads to “to divisive conflict, alienation, and ultimately, poor performance” [82].^31^

### The Criminalization of Dissent: Recent Examples from Spanish Law

Some European governments seem to go further by making criminal laws that prosecute dissenters. Spain has been taking some steps in that direction as a means of guaranteeing national security or defending other rights connected to a broad exercise of sexual freedom. I will briefly present three examples.

In 2015, Article 578 of the Spanish criminal code was broadened in response to the Paris attacks and the perceived threat of international terrorism, although the vast majority of the cases brought under the law related to disbanded or inactive domestic armed groups, namely ETA and GRAPO. An EU Directive on combating terrorism, which included “glorification” as an example of expression that may be criminalized and was implemented across Europe at the end of 2018, led the Spanish government to vaguely define offences such as “glorification of terrorism” and “humiliation” of its victims, seriously endangering the right to freedom of expression. According to Amnesty International, an exponential increase in the number of people falling foul of a draconian law banning the “glorification of terrorism” or “humiliating victims of terrorism” seems to be part of a sustained attack on freedom of expression in Spain [34]. In this vein, it has been stated that “Spain is emblematic of a disturbing trend which has seen states across Europe unduly restricting expression on the pretext of national security and stripping away rights under the guise of defending them.”^32^

Other recent criminal laws restrict the freedom of expression when it is considered to attack the exercise of sexual freedom. The Spanish government took the initiative of drafting a criminal provision, that was issued in April this year and came into force immediately in the Criminal code [93], whereby it is labeled as harassment the fact of being close to hospitals where abortions are performed in order to pray, provide information and offer assistance to those women who approach these hospitals seeking medical support to interrupt their pregnancy. The new provision reads as follows:

1. Whoever, in order to hinder the exercise of the right to voluntary interruption of pregnancy, harasses a woman through annoying, offensive, intimidating or coercive acts that undermine her freedom, will be punished with a prison sentence of three months to one year or work for the benefit of the community from thirty-one to eighty days.
2. The same penalties will be imposed on whoever, in the manner described in the previous section, harasses health workers in their professional practice or public function and the medical or managerial staff of centers authorized to interrupt pregnancy with the aim of hindering the exercise of his profession or position.
3. Taking into account the seriousness, the personal circumstances of the author and the concurrent in the realization of the act, the court may also impose the prohibition to go to certain places for a period of six months to three years.
4. The penalties provided for in this article will be imposed without prejudice to those that may correspond to the crimes in which the acts of harassment took place.
5. In the prosecution of the facts described in this article, the complaint of the aggrieved person or his legal representation will not be necessary.”^33^

One might wonder if there was a need for such provision, particularly when Art. 172. 1 perfectly defined the criminal offense of harassment, whereby those who approached women in an unrespectful manner that constitutes coercion or harassment could perfectly be punished under such provision:

Whoever, without being legitimately authorized, violently prevents another from doing what the law does not prohibit, or compels him to do what he does not want, whether fair or unfair, shall be punished with a sentence of imprisonment from six months to three years or with a fine of 12 to 24 months, depending on the severity of the coercion or the means used.^34^

It seems clear that if the Criminal code already punished anyone who “violently prevents another from doing what the law does not prohibit, or compels him to do what he does not want, whether fair or unfair,” there was not much need for an additional provision, unless the purpose was precisely to restrict the expression of those who dissent from the current law and devote themselves to pray, inform and give support to those women who freely want to receive it. In short, if the behavior of those who pray before the abortion clinics has sufficient intensity to violate the will of others, their actions are already included and punished by criminal law (art. 172.1); however, if their action is not violent (neither physical, nor intimidating, nor with force on things), then we are not facing harassment or coercion. What's more: such a behavior falls within the scope of freedom of expression (art. 16 Spanish Constitution, SC), freedom of assembly (art. 21 SC) and – more in favor of those who pray – their religious freedom and conscience (art. 20 SC) [32].

One might argue that the presence of these people in the nearby of abortion clinics might be annoying, even when they do not behave violently. Yes, that is true. However, is it legitimate to restrict freedom of expression because of such annoyance? Is not praying, informing or expressing disagreement or criticism part of a plural democracy? Can a criminal-law provision consider prayer as an annoying, offensive, intimidating or coercive act?

The defense of some rights might restrict the exercise of free speech indeed, as stated in the Spanish Constitution.^35^ Besides, the Constitutional Court has clearly established that, in the event of a conflict of rights, the principles of weighting, reasonableness and proportionality must be applied [106], and that, in the in the specific case of freedom of expression, the limits are both in the form (no doubt injurious or outrageous phrases and expressions would fit without relation to the ideas or opinions that are exposed and that are unnecessary to expose them), and in the content (that is, the public relevance and the veracity of what is expressed). Freedom of expression is not an absolute right [55, 152–154], but it cannot be restricted on grounds of annoyance, as the Constitutional Court stated it recently:

Freedom of expression includes, along with the mere expression of value judgments, criticism of the conduct of others, *even when it is bland and may annoy, disturb or upset the person it addresses, as pluralism requires it, tolerance and the spirit of openness, without which there is no democratic society*. In the broad framework that is granted to freedom of expression, according to our doctrine, ‘those manifestations that, although they affect the honor of others, are revealed as necessary for the exhibition of ideas or opinions of public interest are protected [107].

In this line of thought, the Administrative Court of Frankfurt am Main recently ruled (16 December 2021) that an order of the public authority that limited when and where the members of a prayer group could congregate contravened the right to freedom of assembly from article 8 of the German Basic Law.

The third example touches upon a ‘Preliminary Bill for the real and effective equality of trans people and for the guarantee of the rights of LGTBI people’[35], that was approved by the Council of Ministers (29 June 2021) in order to follow its established procedure, a draft that would soon be presented and passed in parliament.

A recent report on the this Bill by the General Council of the Judiciary (‘Consejo General del Poder Judicial’) argues that the repeated use of the legislative technique (of resorting to comprehensive and transversal regulations), in addition to overlapping with other current laws, leads to an “excessive atomization of the legal system” by providing certain groups with a privileged regime of protection, differing from the regime applicable to the rest of the citizens, “with notable detriment to the right to equality and the principle of legal certainty.”^36^ This lack of legal certainty affects the exercise of freedom of speech about LGTBI issues, particularly when this law contains a disciplinary system composed of administrative fines for those who, in expressing their views, might infringe LGTBI rights.^37^ In this vein, Art. 76.2 a) considers as a minor infringement “the use or emission of degrading expressions against people because of their sexual orientation and identity.” Will any dissenting opinion about the goodness of LBTBI style of life be considered ‘degrading’? Will this law protect those who express their dissenting thought about this issue? Or will it rather treat those dissenters as discriminators and hence threaten them with administrative sanctions, if they dare to say or write anything that might be considered as an annoyance or as discriminatory?[100].^38^ Such laws that seek to enhance the positive discrimination might not distinguish between the free expression of ideas and the degradation of someone in particular. This is so because any dissenting opinion is erroneously considered as degrading for that particular group of people, so freedom of expression is substantially restricted and threatened by administrative and criminal sanctions.

Who are here the most vulnerable? Those who are being protected by laws like this, or those who will not be able to express what they think (inasmuch as they have a dissenting opinion that cannot be expressed because it is erroneously regarded as a threat towards those at discriminatory risk)? The reader might think about it and judge for him/herself, but perhaps may agree with me that illegitimate restriction of free speech necessarily produces vulnerability of those who are not allowed to express their thought, causing a discrimination that touches upon a necessary requirement of human dignity and a fundamental principle of democracy [2, 7, 25, 27, 30–50, 84].

## Concluding Remarks

Suppose people demand protection from words and ideas they do not like, perhaps because they are not politically correct or because they are against the public morality of a particular moment, and laws do not allow the expression of such views that might cause emotional distress to some people. In that case, we have two different kinds of vulnerability, namely that of those prone to be emotionally distressed and that of those who are not allowed to express those views that are not orthodox or might produce distress to some people. In the end, what happens is that those who are not allowed to express their views, because of the social and legal consequences, become second-rate citizens. They also become more vulnerable (because they are not allowed to cause emotional distress with their opinions, while they cannot claim themselves distressed by the opinions of others), and, more importantly, democracy becomes weaker, less plural, and less inclusive.

Democracies need free and mature societies composed of individuals able to listen to different views about human life, human dignity, and about how to live in society [73]. Otherwise, “[i]t becomes necessary then to question the fragility of intellectual freedom in established democracies, and their vulnerability to censorship. Without a firmly-entrenched culture of intellectual freedom, how can an established democracy claim the moral high ground when it tries to convince an authoritarian state about the perils of censorship?”[32, 37].

Universities should take the lead in creating an environment of intellectual freedom, enhancing an open debate, and broadening the minds of their students, but most of them do not seem to be on the right path. Unfortunately, this is the general tendency of Western universities, not only in the United States but in the Commonwealth [92, 108], Europe, and America. In many universities, expressing a different opinion against some highly ideologically charged issues such as abortion, gender, feminism, marriage, and family is viewed as an intolerable act that deserves to be immediately punished –in the social, professional, and legal domains–, affecting the whole university community, including lecturers,^39^ officers,^40^ and students.^41^

Paradoxically, it is intolerance for the sake of ‘tolerance,’ totalitarianism for the sake of ‘free democracy,’ exclusion for the sake of ‘inclusivism,’ uniformity for the sake of ‘equality,’ pensée unique for the sake of ‘diversity’ and restriction of free speech for the sake of ‘pluralism.’ I can only doubt the consistency and coherence of such ‘tolerance,’ ‘free democracy,’ ‘inclusivism,’ ‘equality,’ ‘diversity’ and ‘pluralism’ when, in the name of such notions, a single-value system is imposed to the whole society and those who dissent are treated as second-class citizens, leaving them vulnerable and also without legal protection.

## Footnotes

1 Article 20 of the current Spanish Constitution enshrines several rights, including the right to “freely express and disseminate thoughts, ideas and opinions by word, writing or any other means of reproduction” (Art. 20.1a). Similar provisions can be found in most modern Western constitutions. The origin of freedom of speech enshrined in the Spanish precept does not differ much from other Western jurisdictions.

2 Alfonso Guerra, the vice-president of Spain from 1982 to 1991, was well aware of this when, after the PSOE won the elections on 28^th^ October 1982, declared that “We are going to develop Spain into such a country that not even the mother who bore it will recognize.” I do not know to what extent it could be accepted that this was the case when the party mentioned above ceased to govern in 1996. However, it is undeniable that the values on which Spanish society was grounded in 1982 had little to do with those four decades later. This may be more or less liked, but it is a fact that today’s society is not the same, nor is it governed by the same public ethics.

3 Modern libertarianism gained a significant recognition in the US academia with Robert Nozick, *Anarchy, State, and Utopia* 76, which was written as a response to John Rawls’ famous book entitled *A Theory of Justice* 81. Some years later, Rawls notably revised his idea on public reason as can been seen in his article “The Idea of Public Reason Revisited” 82: 765–807.

4 Such a liberal and utilitarian conception of morality and law whose foundation is the mere willingness (rather than reasonableness), has also consequences in other fields of law, such as in countering terrorism, as shown by Aniceto Masferrer & Pedro Talavera 61: 44–55.

5 As it was argued by the Committee on Homosexual Offences and Prostitution, 18. On the matter, see Eustace Chesser, 16, as well as debate between P. Devlin and H.L.A. Hart; on that controversy, see Patrick Devlin, 25, 26; Herbert L.A. Hart 43.

6 In a quite different way, Immanuel Kant was also perfectionist, as some of his works reflect 43, 90; see also Kadri Simm 91: 54-62; see also John Finnis 31: 1-26, “whose main thesis could be summarized as follows: “In any sound theory of natural law, the authority of government is explained and justified as an authority limited by positive law (…), by the moral principles and norms of justice which apply to all human action (…), and by the common good of political communities-a common good which I shall argue he is inherently instrumental and therefore limited”.

7 The expression ‘deliberative democracy’ was coined by Joseph Bessette 12, 13: 102–116.

8 Stanley Ingber 45: 1-91: “John Stuart Mill thus argued that repression may interfere with the market's ability to seek truth: first, if the censored opinion contains truth, its silencing will lessen the chance of our discovering that truth; secondly, if the conflicting opinions each contain part of the truth, the clash between them is the only method of discovering the contribution of each toward the whole of the truth; finally, even if the censored view is wholly false and the upheld opinion wholly true, challenging the accepted opinion must be allowed if people are to hold that accepted view as something other than dogma and prejudice; if they do not, its meaning will be lost or enfeebled.”.

9 See also Simon Clarke 17: the author argues that “…democracy is served not only by the abilities that broad freedom of speech helps develop but also by the self-development that would occur under freedom more generally. The connection between democracy and self-development was explored by Mill in chapter 3 of *Considerations on Representative Government*. There he argued that democracy is more likely, compared to other forms of government, to result in the development of intellectual, practical, and moral abilities. Intellectual abilities involving abstract speculation would be developed by people thinking about possible solutions to national problems, with the prospect of putting into effect. Practical abilities, the abilities involved in applying abstract speculations to practical matters, would be developed by the participation in politics that democracy encourages. Moral abilities, involving concern for the interests of others, are furthered by having the ability to make decisions which will affect those interests. In a democracy, people have to think about how political decisions will affect everyone.”.

10 “The Court's supervisory functions oblige it to *pay the utmost attention to the principles characterising a "democratic society". Freedom of expression constitutes one of the essential foundations of such a society, one of the basic conditions for its progress and for the development of every man*. Subject to paragraph 2 of Article 10 (art. 10–2), it is applicable not only to "information" or "ideas" that are favorably received or regarded as inoffensive or as a matter of indifference, but also to those that offend, shock or disturb the State or any sector of the population. Such are the demands of that pluralism, tolerance and broadmindedness without which there is no "democratic society" 41. This means, amongst other things, that every "formality", "condition", "restriction" or "penalty" imposed in this sphere must be proportionate to the legitimate aim pursued”; italics are mine; in a similar way, it was recently stated that “[f]reedom of expression is both an individual human right and a prerequisite for a functioning democracy” (*Mandate for the Freedom of Expression Commission*, 14 February 2020, based on article 100 of the Constitution of the Kingdom of Norway, the Commission is to review the social, technological, legal and economic frameworks for freedom of expression in today's society).

11 “The menace of hate speech is mainly affecting minorities, as approximately 70% of victims of hate speech or hate crimes belong to minorities. However, the brutality and violence of hate speech, stemming from racism, prejudice, scapegoating and incitement to violence in social media is often ignored or normalized, with victims remaining unnamed. This is a critical issue to tackle, as freedom of expression, one of the main pillars of a free, stable and democratic society, is constantly stressed and its restrictions tested. Following these premises, the dark side of social media cannot be ignored anymore, as it has become a vehicle for dangerous forms of hate speech, conspiracy theories, misinformation, disinformation, racism and scapegoating of minorities to conjure and spread” 105:4.

12 “It is usual for a hate speech of a person belonging to a dominant majority group in the society to be more alarming than a hate speech of a person belonging to a vulnerable and discriminated minority. However, hate speech can be targeted by a minority towards another minority or vulnerable group in the society. For instance, hate speech of a person belonging to a discriminated ethnic minority targeted towards a gay community or LGBT population, expression of sexism, chauvinism or misogyny” 11: 31–32.

13 “Most of the time, people are “cancelled” because they are a public figure with influence over a huge audience and what they’ve done or said is alleged to have caused harm to a particular person, group of people or community. For example, many of those who have been “cancelled” have received this public backlash following accusations of violent, sexist, racist, homophobic or transphobic activities or comments” 108.

14 “The concept chilling effect is central to my approach, since this effect has demonstrated its effectiveness in stimulating self-censorship and thus less transparency in any given public sphere, and since the chilling effect may have severe repercussions vis-à-vis this particularly important strand of journalism. Surveillance and the threat of being surveilled (and thus perhaps also persecuted) are in turn important components of the chilling effect…” 28: 227–242, 228.

15 “The concept chilling effect is central to my approach, since this effect has demonstrated its effectiveness in stimulating self-censorship and thus less transparency in any given public sphere, and since the chilling effect may have severe repercussions vis-à-vis this particularly important strand of journalism. Surveillance and the threat of being surveilled (and thus perhaps also persecuted) are in turn important components of the chilling effect…” 28: 227–242, 228.

16 As can be seen, the chilling effect is mentioned in 12 country chapters: Bulgaria; Germany; Estonia; Ireland; France; Croatia; Hungary; Poland; Portugal; Romania; Slovenia; Finland. The Spanish chapter makes no reference about the chilling effect, but states that “in recent years instances of hostility towards journalists have been registered, including situations where journalists were the targets of threats or violence” 100.

17 “I feel that increased government surveillance has had a chilling effect on my research, most of which I do on the Internet. This includes research on issues such as the drug wars and mass incarceration, which people don’t think about as much as they think about foreign terrorism, but is just as pertinent.” 79: 4, 100.

18 The *Report for the United Nations Commission on Human Rights* denounced that “The poverty and social marginalization endured by large sectors of society in the Americas affect the freedom of expression of the hemisphere’s citizens, in that their voices are ignored and consequently left out of any debate” 102.

19 “Vulnerable adults are often ignored in the debate around digital citizenship education. A vulnerable person is someone who is unable to look after themselves, protect themselves from harm or exploitation or are unable to report abuse. This covers a wide range of people, such as those of with physical disabilities or illnesses, neurodiverse individuals, care leavers, people with mental health difficulties, those with addictions, and homeless people” 84.

20 “This exclusion from debate is despite the additional risks that the internet poses to vulnerable people: there is significant scope for harassment, bullying, exposure to harmful content, sexual grooming, exploitation, encouragement of self-harm, and access to dangerous individuals or information” 84.

21 “Creating an inclusive online environment is therefore especially important for vulnerable adults to ensure they can express their views freely online. The Government has identified a lack of support for vulnerable adults as a barrier to improving their literacy” 84.

22 Summary of conclusions and recommendations, n. 24: “Anonymity can allow individuals, including those in vulnerable positions, to express themselves more freely and to challenge orthodoxies. This is crucial. However, it can also embolden people to abuse others.”.

23 Human vulnerability for not being allowed to express one´s views do not affect children, although laws protecting them might justify censorship or limit the scope of the free exercise of expression; on this matter, see Elisabeth Staksrud, Kjartan Olafsson & Tijana Milosevic 73: 159–173.

24 *Mandate for the Freedom of Expression Commission*, 14 February 2020, based on article 100 of the Constitution of the Kingdom of Norway, the Commission is to review the social, technological, legal and economic frameworks for freedom of expression in today's society.

25 *Freedom of expression under pressure*: “In many countries, freedom of expression and media freedom are under severe pressure. The COVID-19 pandemic has exacerbated a negative trend in which democratic values and human rights are increasingly challenged, and autocratisation is accelerating. Many countries, including a number of democratic countries, have introduced new restrictions that limit freedom of expression. Journalists and the media are being prevented from doing their job in various ways, and human rights defenders and other critical voices are being denied access to information and silenced. Government-sanctioned censorship of the internet and media channels is a growing problem. It is essential to strengthen freedom of expression and freedom of the press in order to restore people’s trust in public institutions and in each other, and to promote support for social development based on respect for human rights and democratic values” 101.

26 *Human rights in the digital space*; and it goes on as follows: “A strong, diversified and independent media sector that can provide critical, fact-based journalism, combined with a high level of public media and information literacy, is also vital for protecting freedom of expression and information 101.

27 Safe environment for freedom of expression. 4.1 Online threats; italics are mine; and goes on: “This includes human rights defenders and civil society organisations that play an important role in giving vulnerable groups a voice, for example, indigenous peoples and human rights defenders working to promote indigenous peoples’ rights to land and natural resources” 101.

28 “However, there is a deeper problem with trigger warnings. According to the most-basic tenets of psychology, the very idea of helping people with anxiety disorders avoid the things they fear is misguided. A person who is trapped in an elevator during a power outage may panic and think she is going to die. That frightening experience can change neural connections in her amygdala, leading to an elevator phobia. If you want this woman to retain her fear for life, you should help her avoid elevators 54. But if you want to help her return to normalcy, you should take your cues from Ivan Pavlov and guide her through a process known as exposure therapy. You might start by asking the woman to merely look at an elevator from a distance—standing in a building lobby, perhaps—until her apprehension begins to subside. If nothing bad happens while she’s standing in the lobby—if the fear is not “reinforced”—then she will begin to learn a new association: elevators are not dangerous. (This reduction in fear during exposure is called habituation.) Then, on subsequent days, you might ask her to get closer, and on later days to push the call button, and eventually to step in and go up one floor. This is how the amygdala can get rewired again to associate a previously feared situation with safety or normalcy.”.

29 The author explains how the UK government’s attempt to “prevent” terrorism and extremism in the university sector can be rightly seen as an intolerant threat to academic freedom, replicating many of the discussions already taking place in universities about the need to protect “vulnerable” students from offensive and dangerous ideas. He argues that, while the threat to academic freedom used to come from outside the university, from pressures exerted from governments, from religious institutions who oversaw a particular institution or from the demands of business, today there is a more dangerous threat to academic freedom that comes from within universities, a triumvirate of a relativistic academic culture, a new body of identity-based student activists and a therapeutically oriented university management, all three of which have helped to construct universities as safe spaces for the newly conceptualized “vulnerable student” 98: 71–92.

30 “People in the organization need to feel that, in questioning themselves or making themselves vulnerable, they will not be judged or punished. In other words, they need to feel safe. Leaders create safety by publicly stating their assumption that people are well-intentioned and by overtly ensuring that well-intentioned actions will not lead to punishment. They resist the judgmental tone that diversity discussions so often acquire, by making it clear that mistakes will not impugn anyone’s moral character. Being candid themselves, they also encourage others to be candid. Perhaps most important, such leaders acknowledge their own fallibility in cross-cultural interactions. When they describe publicly their own learning, they legitimate discussions of identity-related experiences, giving permission to employees to provide and solicit feedback, air conflicts, and learn from their missteps” 29.

31 “The five principles we have identified are difficult to enact. They entail taking risks and opening up when we feel most vulnerable and in need of self-protection. When others accuse us of holding prejudicial attitudes, we should interrogate ourselves; when we believe others are treating us unfairly, we should reach out to understand their actions. These prescriptions do not sell easily; self-righteousness feels more satisfying. But self-righteousness can also lead to divisive conflict, alienation, and ultimately, poor performance” 29.

32 Eda Seyhan, Amnesty International and Esteban Beltrán, Director of Amnesty International Spain pointed out as follows: “Sending rappers to jail for song lyrics and outlawing political satire demonstrates how narrow the boundaries of acceptable online speech have become in Spain. (…). People should not face criminal prosecution simply for saying, tweeting or singing something that might be distasteful or shocking. Spain’s broad and vaguely-worded law is resulting in the silencing of free speech and the crushing of artistic expression.

33 Art. 172 quarter, Spanish Criminal Code.

34 Art. 172.1 Spanish Criminal Code.

35 Art. 20.4 Spanish Constitution: “(…) especially, in the right to honor, privacy, one's own image and the protection of youth and childhood”.

36 For a resumé of the Report, see https://www.poderjudicial.es/cgpj/es/Poder-Judicial/En-Portada/-El-Pleno-del-CGPJ-estudiara-el-dia-20-la-propuesta-de-informe-al-anteproyecto-de-Ley-para-la-igualdad-real-y-efectiva-de-las-personas-trans-y-para-la-garantia-de-los-derechos-de-las-personas-LGTBI–.

37 Art. 76.1: “Infringements regarding equal treatment and non-discrimination based on orientation and sexual identity, gender expression or sexual characteristics are classified as minor, severe and very serious, in view of the nature of the unfulfilled obligation and the entity of the right affected.”.

38 Guadalupe Sánchez argues that the Bill establishes “a disproportionate sanctioning system – fines of up to 150,000 euros –, which seeks to administratively limit freedom of expression and condition legal and scientific debate, so that the premises from which the law is based become dogmas of faith unquestionable. That is what has always been called censorship, but this time under the noble alibi of the fight against discrimination. In the near future, writing and publishing articles like this one will be worthy of a sanction if the collectives and trade unions of the day (to whom the preliminary draft confers legitimacy for the exercise of judicial actions in the civil, contentious-administrative and social spheres), consider that contain expressions that can be considered humiliating” 85.

39 There is a countless number of cases, being many of them reported in internet; see, for example, https://nypost.com/2022/04/30/professors-on-how-they-were-canceled-why-they-fought-back/, https://www.nas.org/blogs/article/tracking-cancel-culture-in-higher-education, and https://www.dailysignal.com/2022/01/03/8-college-professors-canceled-by-left/.

40 In the last few months, for example, the Chancellor of the University of Valencia dismissed both Amparo Mañés from her office as Director of the Equality Unit, for her opinions on Queer theory and feminism (see, for example, https://www.elespanol.com/espana/comunidad-valenciana/20220412/universidad-valencia-igualdad-decir-mujer-hembra-humana/664433625_0.html), and Ferran Suay, Director of Languages and Linguistic Policy, for his “unacceptable and sexist” comments (see https://www.abc.es/espana/comunidad-valenciana/abci-universitat-valencia-cesa-director-politica-linguistica-comentarios-machistas-redes-sociales-202206091800_noticia.html).

41 Recently, on June 27, 2022, Christian Fernando Cortés Pérez, an outstanding student –with 99.9 out 100 in his GPA– who gave the commencement address in the Psychology graduation ceremony at the Universidad Autónoma de Baja California (in Ensenada, Baja California, México), is going through a brutal bullying by a lgbtqia + group that wrote a letter to the chancellor asking to deprive him from his university degree and disqualifying him to work as a psychologist for his disrespectful views. Cortés Pérez had just resorted to science to argue for the need to protect the right to life and dissented from the Queer theory that ignores the evidence of biology and natural sciences (for a description of the case, see https://youtu.be/pnGeMP7e3KA).
